# Sequential compression devices in postoperative urologic patients: an observational trial and survey study on the influence of patient and hospital factors on compliance

**DOI:** 10.1186/1471-2490-13-20

**Published:** 2013-04-11

**Authors:** David F Ritsema, Jennifer M Watson, Amanda P Stiteler, Mike M Nguyen

**Affiliations:** 1 Division of Urology, The University of Arizona, Tucson, AZ, USA; 2 Department of Biomedical Engineering, The University of Arizona, Tucson, AZ, USA; 3 Department of Urology, The University of Southern California, Los Angeles, CA, USA

**Keywords:** Venous thromboembolism, Intermittent pneumatic compression devices, Postoperative complications, Compliance

## Abstract

**Background:**

Sequential compression devices (SCDs) are commonly used for thromboprophylaxis in postoperative patients but compliance is often poor. We investigated causes for noncompliance, examining both hospital and patient related factors.

**Methods:**

100 patients undergoing inpatient urologic surgery were enrolled. All patient had SCD sleeves placed preoperatively. Postoperative observations determined SCD compliance and reasons for non-compliance. Patient demographics, length of stay, inpatient unit type, and surgery type were recorded. At discharge, a patient survey gauged knowledge and attitudes regarding SCDs and bother with SCDs. Statistical analysis was performed to correlate SCD compliance with patient demographics; patient knowledge and attitudes regarding SCDs; and patient self-reported bother with SCDs.

**Results:**

Observed overall compliance was 78.6%. The most commonly observed reasons for non-compliance were SCD machines not being initially available on the ward (71% of non-compliant observations on post-operative day 1) and SCD use not being restarted promptly after return to bed (50% of non-compliant observations for entire hospital stay). Mean self-reported bother scores related to SCDs were low, ranging from 1–3 out of 10 for all 12 categories of bother assessed. Patient demographics, knowledge, attitudes and bother with SCD devices were not significantly associated with non-compliance.

**Conclusions:**

Patient self-reported bother with SCD devices was low. Hospital factors, including SCD machine availability and timely restarting of devices by nursing staff when a patient returns to bed, played a greater role in SCD non-compliance than patient factors. Identifying and addressing hospital related causes for poor SCD compliance may improve postoperative urologic patient safety.

## Background

Venous thromboembolism (VTE) is a significant safety issue among all hospitalized patients, resulting in considerable morbidity, mortality, and financial burden to the patient and to the health care system. This risk is higher for those patients who undergo urologic procedures involving pelvic organs for cancer such as prostatectomy or cystectomy [1]. Because of the high prevalence of VTE among hospitalized patients, the adverse consequences of VTE, and the efficacy of thromboprophylaxis, preventative measures against VTE are routine practice in most health care facilities to improve patient safety [2].

Options for prophylaxis include pharmacologic anticoagulation or mechanical thromboprophylaxis. Mechanical methods are commonly favored for patients at high risk for bleeding, such as post surgical patients, because properly administered mechanical methods have equivalent prevention of thrombotic events with decreased risk of major bleeding [3]. In a study comparing the use of heparin versus the use of a mobile compression device following total hip arthroplasty, major bleeding events occurred in 6% of patients in the heparin group while there were no major bleeding events in the compression device group [4]. In light of these data, many surgeons have adopted mechanical methods as the standard for VTE prophylaxis in hospitalized post-operative patients.

In spite of the known benefits of proper SCD use, studies have demonstrated that SCD compliance is generally low, with devices applied and properly functioning only on average of 60% of the total time that the patients were monitored in one study [5], and 48% of the overall time in another [6]. The factors contributing to inadequate use of SCDs may originate from any or all components of the medical system including patients, nurses, physicians, medical supply departments, hospital administration/policy, and the devices themselves.

Prior research and interventions for improving SCD compliance have primarily focused on hospital staff factors and used training and education to increase knowledge among health care providers and nursing staff [7,8]. These approaches have demonstrated limited success, suggesting that other unrecognized hospital or patient factors may have greater influence over SCD compliance. In this study we sought to evaluate all potential factors that may contribute to inadequate use of SCDs. Identifying which other factors are at fault may help guide improved interventions to improve SCD compliance and safety among postoperative patients.

## Methods

All study protocols were reviewed and approved by the University of Arizona Medical Center IRB.15 hospital staff and 15 patients were initially interviewed as key informants to elicit concepts regarding knowledge, attitudes, and beliefs regarding SCDs and their use, focusing on perceived reasons for poor compliance. Key informants consisted of post-operative urologic patients who had experienced the use of SCDs and nurses or patient care technicians who worked with postoperative patients. This information was used to develop a patient survey.

The patient survey was used to gauge patient experience with and attitudes towards SCDs and allowed their observations to reflect hospital factors such as nursing behavior, physician behavior, and hospital availability of SCDs as well as bother with SCDs. Major survey topics included understanding of the purpose of SCDs, experience and perceptions regarding the application and availability of SCDs, perceptions regarding bothersome and pleasurable aspects of SCDs, beliefs about why SCDs are not worn all the time, and suggestions for improvement of SCDs. Recorded demographics included age, gender, education, and surgery performed. The survey was first pilot tested with 10 non-study patients to assess readability and understanding of the questions. The final survey is shown in Additional file 1.

A total of 100 patients undergoing inpatient urologic operations were then enrolled in the study protocol. Subjects were eligible if they were admitted as an inpatient, older than 18 years, were literate in English, and were able to provide informed consent. Subjects were excluded if they were missing lower extremity limbs precluding use of SCDs, or had an active pulmonary embolism/deep venous thrombosis. Each subject received SCD leg sleeves upon entering the operating room. On post-operative day 1, all subjects were verbally informed once by resident housestaff that SCDs helped prevent blood clots and that they should always remain in place and powered-on while the patients were in bed. For the duration of each subject’s hospital stay, twice-daily observations by research staff were performed to determine compliance, and, in cases of non-compliance, the cause of non-compliance. The percentage of compliant observations for each individual was recorded. Recorded reasons for device non-compliance included non-availability of devices, non-function of devices, device removal by patients or nursing staff, failure to replace device after removal, or other cause. SCD use was considered compliant if SCD leg sleeves were in place and the machine was turned on while a patient is in bed. SCD use was also considered compliant if SCDs were not in place but subjects were ambulating, sitting in a chair, or transferring in or out of bed. Length of stay, type of inpatient unit (intensive care versus ward), and diagnosis of pulmonary embolism or deep venous thrombosis were also recorded. Subjects were blinded to the objective of the study until immediately prior to their discharge, at which time they were given the study survey. Because of non-response by 3 subjects to the survey, 3 additional subjects were recruited to reach a total of 100 subjects with data from surveys and observations.

Summary statistics were constructed with means for continuous variables and percentages for categorical variables. Chi-squared tests were used for analysis of dichotomous variables and t-tests for continuous variables. Statistical analysis was performed to correlate SCD compliance, the primary outcome of the study, with patient demographics, self-reported bother with SCDs, knowledge of SCDs, and attitudes towards SCDs. The mean percentage of compliant observations for each subgroup was determined and t-tests or analysis of variance tests were used to compare percentage compliance between groups to determine significant differences. Because of the non-normal distribution of self-reported bother scores, Spearman’s rank correlation was used to evaluate the correlation between self reported bother and compliance. Analysis was performed using the STATA 10 statistical software package (Stata Corporation, College Station, Texas). Statistical significance was set at 0.05 and all tests were two-tailed. No funding was used for this study. University of Arizona IRB approval was obtained for this study.

## Results

### Overall characteristics

Surveys were completed immediately prior to discharge by 100 patients who underwent a urology procedure requiring an inpatient stay. The mean age was 58.6 years. Gender was 76% male and 24% female. Educational status included 23% completing high school or less, 58% completing college or some college, and 18% completing a masters or doctorate degree. Types of surgical operations that patients underwent were divided into categories of open (29%), laparoscopic/robotic (27%), endoscopic (17%), and female pelvic and male urethral surgeries (27%). Length of stay was 1 day for 41% of patients, 2–5 days for 47%, and 5–9 days for 12%. One patient developed a deep venous thrombosis during his hospital stay. He was an 81-year-old male who underwent an open cystectomy procedure associated with an 8-day hospital stay. His observed SCD compliance during his hospital stay was 93%. Compliance results evaluated by individual characteristics are summarized in Table 1.

**Table 1:** Compliance by individual characteristics

**Characteristic**	**Mean compliance**	**95% confidence interval**	**p value**	**Test**
**Gender**			0.8697	t test
Male	79.7%	72.7%-86.6%		
Female	78.5%	65.7%-91.3%		
**Age category**			0.3635	ANOVA
<49 yo	77.2%	64.2%-90.1%		
50-59 yo	72.1%	59.7%-84.5%		
60-69 yo	86.2%	76.9%- 95.4%		
70+	81.6%	68.1%-95.2%		
**Education**			0.6486	ANOVA
High school or less	78.4%	66.6%-90.2%		
Some college/college graduate	82.4%	75.3%-89.4%		
Masters/doctorate	75.5%	58.1%-92.8%		
**Surgery type**			0.7281	ANOVA
Open	77.0%	69.1%-85.0%		
Lap/robotic	84.9%	75.1%-94.7%		
Endoscopic	79.4%	60.7%-98.1%		
Female pelvic/male urethral	76.4%	62.5%-90.4%		
**Length of stay**			0.4639	ANOVA
1 day	84.2%	72.3%-96.1%		
2-3 days	76.4%	67.8%-85.0%		
4-9 days	76.5%	67.7%-85.3%		

### Research staff observations

**Figure 1:**
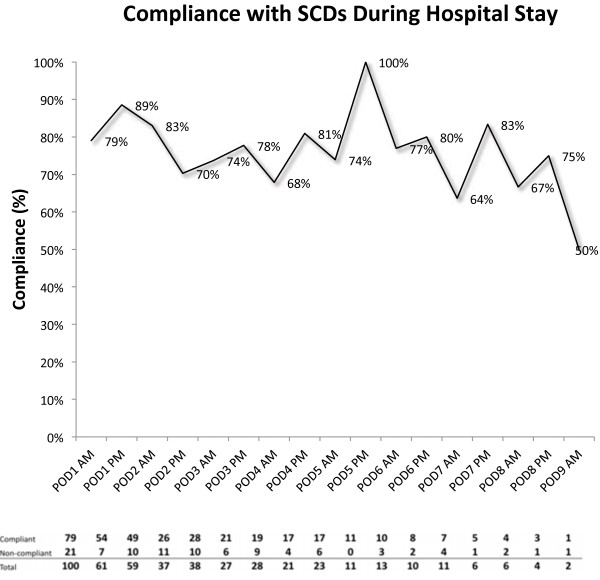
Mean compliance at each observation time.

A total of 457 observations were made among all patients. Observations for compliance revealed 359 compliant observations and 98 non-compliant observations, giving an overall compliance of 78.6%. Compliance did not demonstrate a consistent trend by day of observation and ranged from 50% to 100% (Figure 1). The single occurrence of 50% compliance was seen on the last observation time point of postoperative day nine when there were just two subject observations available. Mean compliance did not vary significantly by age group (77% for <49 y.o., 72% for 50–59 y.o., 86% for 60–69 y.o., 82% for 70+ y.o.; p=0.3635); gender (79% for females and 80% for males; p=0.8697); education (78% for high school or less, 82% for some college or college graduate, 76% for more than college; p=0.6486); type of operation (77% for open, 85% for laparoscopic/robotic, 79% for endoscopic, and 76% for female pelvic and male urethral surgeries; p=0.7281); or length of stay category (82% for one day, 82% for 2–3 days, 73% for 4–9 days; p=0.3765) (Table 1).

The most common reason for non-compliance on the first observation in the morning on post-operative day 1 (21 non-compliant observations) was that no machine was present (71%); followed by the SCDs not being replaced (19%); SCDs bothering or being uncomfortable (5%); and the machine not being turned on or restarted (5%) (Table 2). When considering the entire hospital stay, the reasons for the 98 non-compliant observations included that the SCDs were not replaced when the patient returned to bed (50%); there was no machine or cuffs available (22%); the SCDs bothered or were uncomfortable for the patient (19%); the machine was not turned on or restarted despite the sleeves being in place (8%); or unknown reasons (1%).

**Table 2:** Reported reasons for non-compliance

		
**Reasons for non-compliant observations, postoperative day 1**		
Reason	n	%
No machine/cuffs	15	*71.4*
Not replaced	4	*19.0*
SCDs bothered the patient/uncomfortable	1	*4.8*
Machine wasn’t turned on/restarted	1	*4.8*
Total	21	*100*
**Reasons for non-compliant observations, all days**		
Reason	n	%
Not replaced	50	*50.0*
No machine/cuffs	22	*22.0*
SCDs bothered the patient/uncomfortable	19	*19.0*
Machine wasn’t turned on/restarted	8	*8.0*
Unknown	1	*1.0*
Total	100	*100*

### Patient surveys

Subjects were consistently able to recall physician instruction given on postoperative day one regarding the purpose of SCDs. Prevention of blood clots was identified as the correct purpose by 99% of patients on their discharge survey and only 16% of patients reported that they would have liked more information on the purpose of SCDs. Most patients also felt adequately educated regarding the application and usage of SCDs, with only 20% desiring more information about how to use the SCDs. Based on the instruction that they received, 92% of patients agreed that the SCDs were important to wear when in bed and at all times unless ambulating. Compliance did not vary significantly with patient knowledge of the purpose or use of SCDs or desire for more information on the purpose or use of SCDs.

Availability and timely replacement of SCDs had the largest impact on compliance based on patient self-reports. There was significantly lower compliance in patients who reported that their SCDs were not available throughout their hospital stay (p= 0.0008) and in patients reporting a delay in replacement of their SCDs of 30 minutes or greater after returning to bed (p=0.0078). These findings were consistent with the research team observations, which identified non-replacement of SCDs and unavailability of SCDs as the most common reasons for SCD non-compliance.

Patients’ self-reported bother from SCDs was separated into 12 categories including SCDs being noisy, painful, confining, sweaty, hot, itchy, time consuming, too tight, or causing insomnia or waking from sleep, tripping or falling, skin irritation, or foot numbness. Average bother scores were low, ranging from 1–3 out of 10 for all categories (Table 3). The highest mean bother score of 3.1 out of 10 was for the category “The SCDs were confining”. In contrast, most patients appeared to enjoy the SCDs, reporting that “The SCDs felt like a massage” with a mean score of 3.9 out of 5 and “The SCDs were comfortable” with a mean score of 3.7 out of 5, with 5 representing “strongly agree”. Self-reported bother score or preference score was not significantly associated with percent compliance for any category by Spearman’s rank correlation. Consistent with the low reported bother scores, only 8% of patients indicated a preference to receive chemical prophylaxis using an injection instead of SCDs, if they were given a choice.

**Table 3:** Summary of patient reported bother with sequential compression devices

**Question**	**Mean**	**95% confidence interval**	p value*
***Range 1-not bothersome to 10-very bothersome***			
The SCDs were noisy	1.3	1.1-1.6	0.8683
The SCDs caused pain	1.4	1.1-1.7	0.6521
The SCDs were confining	3.1	2.6 - 3.6	0.7058
The SCDs caused sweating	1.8	1.4-2.1	0.722
The SCDs made my legs hot	1.9	1.5-2.2	0.7509
The SCDs caused insomnia or waking from sleep	2.1	1.6-2.5	0.1514
The SCDs caused tripping or falling	1.4	1.1-1.7	0.1693
The SCDs caused skin irritation	1.3	1.0-1.5	0.602
The SCDs caused leg or foot numbness	1.5	1.2-1.8	0.6193
The SCDs were itchy	1.7	1.3-2.2	0.4069
Putting on or taking off the SCDs was time consuming	1.7	1.4-2.0	0.1804
The SCDs felt too tight	1.8	1.4-2.2	0.7146
***Range 1-strongly disagree to 5-strongly agree***			
The SCDs felt like a massage	3.9	3.7-4.2	0.2731
The SCDs were difficult to use	1.3	1.1-1.4	0.9715
The SCDs were comfortable	3.7	3.4-3.9	0.7915

*p value indicates Spearman’s rank correlation between question response and observed compliance.

From the patients’ perspective, the three most important perceived barriers to SCD compliance included “SCDs prevented walking or getting up” (47%), “SCDs were tethering or tangling” (25%) and “SCDs woke the patient from sleep” (15%). When asked how the SCDs could be improved, patients most commonly answered that the devices should be wireless or cordless (49%). Sixteen percent of patients felt that SCDs should have lighter weight/cooler material. Thirty-five percent of patients felt there was no need for improvement. When asked, many patients felt that even if they were taught how to use the SCDs themselves, they would be unable to do so either because of physical limitations (40%) or the complexity of the device (2%), reiterating that hospital staff are key for SCD device compliance.

## Discussion

Sequential compression devices, while effective at reducing the incidence of venous thromboembolism when properly used, often have poor rates of compliance. Overall compliance in our patient population of urologic postoperative inpatients was 78.6%. This was comparable to an intensive care unit compliance rate of 78% found by Comerota but was higher than the 48% compliance seen on routine nursing units in the same study [6]. We found that hospital factors, including availability of SCDs and timely replacement of SCDs when a patient returns to bed, were the primary factors in non-compliance. Patient factors including demographics, knowledge, attitudes, and bother with SCDs did not play an important role in non-compliance.

Other studies have also suggested hospital related reasons for poor SCD compliance. Cornwell et al. identified that in 95% of instances of non-compliance, SCD devices were not in place [9]. Comerota and colleagues identified a main reason for non-compliance in their study to be SCD device pumps not running despite appropriate sleeve placement [6]. Failure to restart SCD devices after treatment was interrupted was identified as the most common cause of non-compliance in a third study [10].

Having medical devices readily available is the most basic requirement for safety device utilization [11,12] but unfortunately was a major cause of non-compliance in our study. As a result of these findings, our facility has obtained additional SCD machines that are left at each patient bed, insuring that the devices will always be available. While necessary, availability alone may not always be sufficient for optimal utilization. For example, introduction of alcohol based hand rubs alone was not sufficient for improved hand hygiene in a study involving three hospital wards. Additional support by medical leadership and a behavioral modification program were also necessary for sustained success [13]. In the case of SCDs, in addition to insuring availability, changing nursing behavior to encourage prompt replacement of devices after a patient’s return to bed may also be necessary in order to adequately address the problem of poor SCD compliance.

Improving medical device design to encourage and simplify use can be another approach to promote compliance [14,15]. In our study, improving SCDs by making them “cordless” or “wireless” was the most common suggested design change to improve SCD compliance, with 49% of subjects listing this when asked about ways in which SCDs could be made easier to use. Such a device would also eliminate the need to remove and replace SCDs when a patient leaves the bed, which was an important cause of non-compliance in our study. Compact, portable, battery-powered pneumatic compression devices that do require constant attachment to a power source have been previously assessed in a study by Murakami et al. [16] In their study, overall compliance was greater in patients using the compact portable devices (78%) compared to patients using traditional SCD devices that required being plugged in at all times and which were bulkier (59%). The difference in compliance was primarily attributed to the ability of the portable devices to continue to operate during patient transport to radiologic procedures. However, the compact portable devices used in their study aren’t entirely self-contained in that they still have tubing connecting sleeves on the patients’ legs to the pump unit. Development of completely self-contained SCDs would likely further improve patient tolerability and compliance.

The acceptability of a therapy to a patient can play an important role in compliance. Easing use and reducing patient inconvenience has been shown to improve compliance with birth control regimens [17] and with continuous positive airway pressure for sleep apnea [18]. Improving patient comfort with SCDs has been proposed as a way to improve compliance [19]. However, our study suggested that patient related factors seemed to play only a minimal role in SCD non-compliance. Out of 12 categories of bother, the highest level of bother reported by subjects in this study was only 3.1 out of 10 for the category “The SCDs were confining.” Patients instead appeared to actually enjoy SCDs, with most reporting that “The SCDs felt like a massage” or “The SCDs were comfortable.” Consistent with these findings, our patients reported higher acceptability to using SCDs over the option of daily subcutaneous anticoagulation shots. Similarly, Cindolo and colleagues found an overall positive opinion about SCDs with 72% percent of patients regarding SCD sleeves as pleasant and 79% reporting that they did not feel oppressive [20].

Several limitations of the study should be noted. We did not use a validated questionnaire to assess patient response. Limited observation time points occurring only twice a day may have weakened the compliance data. Continuous patient monitoring would have been optimal but was not logistically feasible for this study. Interactions with subjects by research staff during observations for compliance may have elevated observed compliance rates. During observations, patients were sometimes asked why they were not wearing their SCDs in order to clarify the cause for non-compliance. This may have encouraged subjects to wear their SCDs more consistently. This study was conducted at one site and findings may not reflect the experience of other medical centers. Additionally, the patients in this study were all postoperative urology patients, and their experiences may not reflect that of other patient populations.

## Conclusions

Patient self-reported bother with SCD devices was low. Hospital factors, including SCD machine availability and timely restarting of devices by nursing staff when a patient returns to bed, played a greater role in SCD non-compliance than patient factors, including patient demographics, knowledge, attitudes, and bother with SCD devices. Identifying and addressing hospital related causes for poor SCD use compliance may improve postoperative urologic patient safety.

## Abbreviations

SCD: Sequential compression device; VTE: Venous thromboembolism.

## Competing interests

The authors declared that they have no competing interest.

## Authors’ contributions

DR conceived of the study, participated in the study design, conducted subject interviews, performed study observations, and drafted the manuscript. JW participated in the design of the study, conducted subject interviews, performed study observations, and helped to draft and revise the manuscript. AS participated in study observations and drafting and of the manuscript. MN participated in the design of the study, performed the statistical analysis, and helped to draft and finalize the manuscript. All authors read and approved the final manuscript.

## Pre-publication history

The pre-publication history for this paper can be accessed here:

http://www.biomedcentral.com/1471-2490/13/20/prepub

## Supplementary Material

Additional file 1 Study exit survey. Click here for file
