# Supplementary material for: Sequential compression devices in postoperative urologic patients: an observational trial and survey study on the influence of patient and hospital factors on compliance
Source: BMC Urol. 2013 Apr 11;13:20. doi: 10.1186/1471-2490-13-20 (PMC3635967; doi:10.1186/1471-2490-13-20)
Supplement: Additional file 1 — Study exit survey. [file 1471-2490-13-20-S1.pdf]

Age: ______________ Gender: ______________

Your highest education level/degree: (check)

☐ Less than high school ☐ High school/GED ☐ Some college ☐ College ☐ Masters ☐ Doctoral

Operation you had: ____

Please mark the response(s) below that best describes your experience with the Sequential Compression Devices (SCDs) that were placed on your legs after surgery.

KNOWLEDGE AND UNDERSTANDING OF SCDS

1. I understood the purpose of the SCDs .... ☐ Yes ☐ No
2. My physician told me about the purpose of the SCDs .... ☐ Yes ☐ No
3. My nurse told me about the purpose of the SCDs .... ☐ Yes ☐ No
4. I would have liked more information on the purpose of the SCDS........ ☐ Yes ☐ No
5. The nurses and physicians told me when the SCDs should be on .... ☐ Yes ☐ No
6. My nurse instructed me on how to use the SCDs .... ☐ Yes ☐ No
7. I would have liked to receive more instruction on how to use the SCDs........ ☐ Yes ☐ No

8. Based on the instruction and attention I received regarding the SCDs, I feel that the SCDs:

Choose one answer.

a) ☐ Were not important to wear during my hospital stay
b) ☐ Were somewhat important but not a priority
c) ☐ Were important only during the first day.
d) ☐ Were important only when I was in bed.
e) ☐ Were important to be worn at all times unless I was walking.

9. To my understanding, the purpose of SCDs is to:

Choose one answer.

a) ☐ Prevent muscle cramps
b) ☐ Prevent blood clots
c) ☐ Assist with relaxation/massage
d) ☐ Prevent bed sores

APPLICATION OF SCDS

10. The SCDs were available immediately following surgery........ ☐ Yes ☐ No ☐ Not sure
11. The SCDs were available throughout my hospital stay........ ☐ Yes ☐ No ☐ Not sure
12. The nurses checked that the SCDs were on and working.... ☐ Always ☐ Usually ☐ Rarely ☐ Never
13. The nurses replaced the SCDs if they were not on........ ☐ Always ☐ Usually ☐ Rarely ☐ Never

14. As best as I can remember, the amount of time that I wore the SCDs during my hospitalization was:
    a) ☐ Less than half of the time I was in bed
    b) ☐ At least half of the time I was in bed
    c) ☐ All of the time I was in bed

15. If I was taught how to put on and take off the SCDs by myself:
    a) ☐ I would be able to operate the SCDs.
    b) ☐ I would not be able to operate the SCDs because they are too complicated.
    c) ☐ I would not be able to operate the SCDs because I couldn't bend or reach down to my legs after my surgery.

16. The delay in replacing the SCDs after I returned to bed was generally:
    a) ☐ No delay
    b) ☐ Less than 10 minutes
    c) ☐ 10-29 minutes
    d) ☐ 30-59 minutes
    e) ☐ 60 minutes or more

BOTHERSOME ASPECTS OF SCD USE

Please circle on the scale from 1-10 how bothersome each of the items listed below were to you:

(1 is not bothersome and 10 is very bothersome)

	Not bothersome	◄							►	Very bothersome
17. The SCDs were noisy	1	2	3	4	5	6	7	8	9	10
18. The SCDs caused pain	1	2	3	4	5	6	7	8	9	10
19. The SCDs were confining	1	2	3	4	5	6	7	8	9	10
20. The SCDs caused sweating	1	2	3	4	5	6	7	8	9	10
21. The SCDs made my legs hot	1	2	3	4	5	6	7	8	9	10
22. The SCDs caused insomnia or waking from sleep	1	2	3	4	5	6	7	8	9	10
23. The SCDs caused tripping or falling	1	2	3	4	5	6	7	8	9	10
24. The SCDs caused skin irritation	1	2	3	4	5	6	7	8	9	10
25. The SCDs caused leg or foot numbness	1	2	3	4	5	6	7	8	9	10
26. The SCDs were itchy	1	2	3	4	5	6	7	8	9	10
27. Putting on and taking off the SCDs was time consuming	1	2	3	4	5	6	7	8	9	10
28. The SCDs felt too tight	1	2	3	4	5	6	7	8	9	10

ADDITIONAL QUESTIONS

Please circle the most appropriate number, corresponding to the statements below:

(1 is strongly disagree and 5 is strongly agree)

	Strongly disagree ⟷				Strongly agree
29. The SCDs felt like a massage	1	2	3	4	5
30. The SCDs were difficult to use	1	2	3	4	5
31. The SCDs were comfortable	1	2	3	4	5
32. If given the choice, I would prefer an anti-clot injection under my skin 2-3 times daily instead of using the SCDs.	1	2	3	4	5

What do you believe are the main reasons why SCDs are not worn all the time?

Place an X next to any answer that applies. If your answer(s) are not on the list, fill in the "other" line.

— Too hot/sweaty
— Uncomfortable
— Noisy
— Wakes me up
— Itching
— Device not available
— Tethering/tangling
— SCDs not replaced after removal
— Difficult to use
— They are not important
— Prevents walking/getting up
— Not enough instructions given
— Other(s) ______________________

What would make SCDs easier to use?

Place an X next to any answer that applies. If your answer(s) are not on the list, fill in the "other" line.

— Lighter weight/cooler material
— Wireless/cordless device
— Better instruction about purpose
— Better instruction about how to use
— No need for improvement
— Family member training to help put on
— Better availability of nurses to assist with the SCDs
— Other(s) _____________________

Please provide any additional comments regarding the SCDs in the space below. Thank you for your time and consideration in completing this survey:
